# Proposal of a Real-Time Test Platform for Tactile Internet Systems

**DOI:** 10.3390/s22249865

**Published:** 2022-12-15

**Authors:** Pedro V. A. Alves, Patricia D. M. Plentz, Marcelo A. C. Fernandes

**Affiliations:** 1 Laboratory of Machine Learning and Intelligent Instrumentation, Federal University of Rio Grande do Norte, Natal 59078-970, Brazil; 2 Graduate Program of Computer Science, Federal University of Santa Catarina, Florianópolis 88036-610, Brazil; 3 Department of Computer Engineering and Automation, Federal University of Rio Grande do Norte, Natal 59078-970, Brazil

**Keywords:** tactile internet, real time, tactile glove, phantom omni

## Abstract

This work aimed to develop a real-time test platform for systems associated with the tactile internet area. The proposal comprises a master device, a communication channel and a slave device. The master device is a tactile glove (wearable technology) that works as a tactile interface based on vibratory feedback. The master device can interact with virtual elements (local or remote). The Matlab/Simulink environment and a robotics toolbox form the communication channel and the slave device. The communication channel introduces a bidirectional connection of variable latency, and the slave device is defined as a robotic phantom omni manipulator emulated in Matlab/Simulink. The virtual robotic manipulator, the slave device, can generate different types of tactile sensations in the tactile glove, that is, in the master device. The platform can model tactile sensations such as coarse roughness, fine roughness, smoothness, dripping and softness. The proposed platform presented adequate results and can be used to test various algorithms and methods correlated to the tactile internet.

## 1. Introduction

In the tactile internet context, embedded systems are defined as emerging technologies [1,2,3,4]. They are divided in three main elements: master device (MD), network and slave device (SD), and the operation modes can vary between teleoperation and machine to machine (M2M). Human master and slave robot is an example of the first operation mode. On the other hand, master robot and slave robot are examples of the second operation mode [5,6,7].

The network’s infrastructure turns possible to transmit tactile sensations between master and slave devices. The communication from master to slave device is direct, and the data sent through the network could be the tactile glove position. Exchanging information from the slave to master device is called as feedback communication, and it’s responsible for tactile data transmission according to [1,2,3,6,7]. Usually, the latency of information exchange among tactile system’s elements needs to be between 1 ms and 10 ms, and 100 ms in some specific cases. Regards to this essential criterion is present in [6,7,8,9].

A discussion about the main aspects of tactile internet and its future prospects is described at [10]. Tactile internet will provide haptic interactions and will allow the transmission of various tactile data through networks. Some potential applications examples are the telemedical procedure, autonomous driving and extended reality. Although the several promising ways to use tactile internet, it is important to develop useful platforms for test applications.

This paper aims to present the development of a real-time test platform for tactile internet. Its three main elements are MD, channel and SD. In this project, the MD is a wearable technology, a tactile glove that acts as an interface based on vibration feedback. This feedback is generated by micromotors, responsible for making possible the interaction with virtual or remote objects. The SD is a robotic phantom omni manipulator inside of the Matlab Simulink environment through the robotics toolbox. The touch of the robotic manipulator in pre-defined positions of a plane represents contact with different textures objects. In this way, the corresponding tactile sensations are generated in the MD. The insertion of tactile stimulus proposed by this platform, together with the visual response, are capable of making the interaction experience more realistic. The main contributions of this article are described as follows:

- present a real-time test platform development for tactile internet applications;
- allow tactile interaction between a human operator, using a tactile glove, with different textures regions simulated on the Matlab-Simulink;
- simulate tactile sensations through vibratory feedback;
- enable the test of various algorithms and techniques associated with tactile internet;
- contribute to the development of new strategies used to minimize problems associated with tactile internet.

The article is organized as follows: the next section will describe some main papers related to advances in tactile internet concepts and real-time tactile platforms. The Section 3 will present the proposed real-time tactile platform, which is capable to manage bidirectional communication between master and slave tactile devices. The results are shown in the Section 4 and the conclusions are presented at Section 5.

## 2. Related Works

Tactile internet is a new field of research. Many works present different platforms, tactile devices, contexts and results. We summarize the main proposals highlighting the contributions of each one.

Medical area explores tactile internet as a way to increase the reach of medical care. In [11], a simulation platform is presented for medical palpation with touch feedback. The master device is configured as a glove capable of receiving tactile feedback on index finger tip through a piezoelectric actuator. A Phantom robotic manipulator is used as a slave device and responsible for touch patients sending information about possible lumps in their body. The tactile platform proposed in this paper differs from the previously mentioned platform for medical palpation due to the kind of feedback used.

In [12] the potential to enable remote surgery is introduced through a specific use case (cholecystectomy) which is mapped to IEEE P1918.1 standard, responsible for making tactile internet connections possible. Ref. [13] features Radiofrequency Augmentation Devices (R-FADs), which are assistive tools in charge of helping people with sensory impairments. The operation takes place through a wrist reader and a fingertip sensor that make it possible for users to feel contact with objects.

In the field of virtual reality technologies, two articles receive attention. The first one [14] seeks to develop a tactile glove with temperature and vibration feedback responsible for simulating human skin tactile perception. The second one tries to demonstrate the importance of adding tactile stimulation in virtualized environments [15] to assist patients in motor recovery with damages caused by stroke. The glove is based on vibration feedback. Both papers propose to integrate virtual reality systems with tactile gloves.

Considering the use of the artificial intelligence, three papers could be cited. Ref. [16] presents the use of artificial intelligence to recognize objects touched by tactile sensors. Object recognition uses a tactile glove with small tactile sensors scattered throughout the glove. In this case, contact points are similar to those present in human hands. Similarly, ref. [17] introduces a tactile glove designed to detect changes in human hand intensity when grasping objects, in order to identify them. That project seeks as a result the detection system application in robots with tactile feedback and in smart prosthesis. Artificial intelligence techniques such as prediction are introduced in [18], to compensate for delay in the control of tactile devices and improve real-time operation perceptions, making the tactile feedback more natural.

In a similar way presented in this project, the papers [19,20,21,22] capture joint movements through an inertial measurement unit (IMU) sensor. The use of this component helps to improve the scalability at low cost because it is possible to increase the number of monitored joints without making the project too expensive. In [23], it is described a more complex way of measuring movement with the use of a camera. The use of a Leap Motion devices to detect rotations in wrist and elbow is presented in [11].

Article [24] is the closest to the one proposed in this work. In that paper, tactile sensations were developed based on vibratory stimulus using PWM signals and changing the duty cycle, high pulse time, frequency and period parameters [24]. These stimulus were generated based on the voltage used in vibration micromotors. The tactile device was used to validate tactile sensations developed through an experiment with users based on semantic differential evaluation method. The outcome of that research is a realism degree associated with each tactile sensation. In a different way, this proposed article seeks to develop a functional platform capable of emulating different types of sensations, enabling algorithms test and techniques associated with tactile internet.

The next section presents the proposal architecture which involves eight software and hardware entities. Together, they deliver a real-time tactile platform for test in a wide range of applications.

## 3. Proposal Architecture

This section presents the proposal architecture for a real-time tactile platform. First, we describe the overall architecture with the main elements and their attributes. Then, we depth the understand about the master device and the hardware attached to it. Communication channel and simulation environment are the next elements described in details. We finalize this section with the presentation of the tactile sensations through the explanation about vibratory feedback and PWM signals.

### 3.1. Overall Architecture

**Figure 1:**
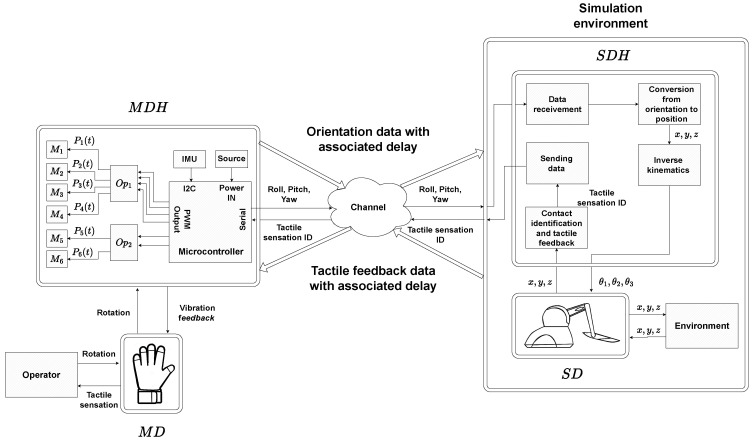
Proposed tactile platform architecture.

The general architecture of the proposed tactile platform is illustrated in Figure 1 and has eight fundamental entities:

1. operator;
2. master device (MD);
3. master device’s hardware (MDH);
4. channel;
5. simulation environment;
6. slave device’s hardware (SDH);
7. slave device (SD);
8. environment.

The left side of the Figure 1 shows the hardware and software components related to the master device (MD). A human operator controls a tactile glove that represents the master device. The tactile glove is based on vibratory feedback and communicates with the master device hardware (MDH). The MDH is formed by an electronic circuit and it is responsible for sending and receiving data from the other elements of the architecture. In other words, it is responsible for the MD’s operation.

In the middle of the Figure 1, it is possible to see the entity channel. It has an important role in the proposed platform because it simulates the real low latency delay between master and slave device. The channel delays the bidirectional communication between the master device hardware and simulation environment.

The right side of the Figure 1 presents the simulation environment entity composed of software device hardware (SDH) and robotic manipulator phantom omni. Both of them work as essential tools to replicate the MD movements performed by the operator in an environment that can be virtual or remote.

### 3.2. Master Device (MD) and Master Device’S Hardware (MDH)

The platform operation cycle begins with the human operator wearing the tactile glove and executing hand movements. In the MDH, these movements are converted to data rotation and send to the simulation environment through the channel. The hand movements represent the exploration process of the slave device (MD). On the other side, this data rotation is received and converted in movement in the slave device phantom omni. After that, a vibratory feedback is generated and sent back to the MDH. The operation cycle ends with the vibratory feedback received at the tactile glove. The vibratory feedback simulates the tactile sensation (*s*) related to the texture touched by the phantom omni.

The MDH is composed of 6 micromotors ($M_{1},M_{2},\cdots ,M_{6}$), 2 optocouplers ($Op_{1},Op_{2}$), an IMU, a battery, and a microcontroller. The arrival of vibratory feedback in MD occurs through PWM signals expressed as ($P_{1}\left(t\right),P_{2}\left(t\right),\cdots ,P_{6}\left(t\right)$) generated by the microcontroller, which arrive at the micromotors ($M_{1},$$M_{2},\cdots ,M_{6}$) after the isolation generated by the optocouplers ($Op_{1},Op_{2}$) to the control circuit (microcontroller) and power (micromotors). The IMU used together with the microcontroller has the function of capturing the rotation performed by the MD in the form of Euler angles. With this, it allows the sending of this data through a serial port to external entities from MDH.

**Figure 2:**
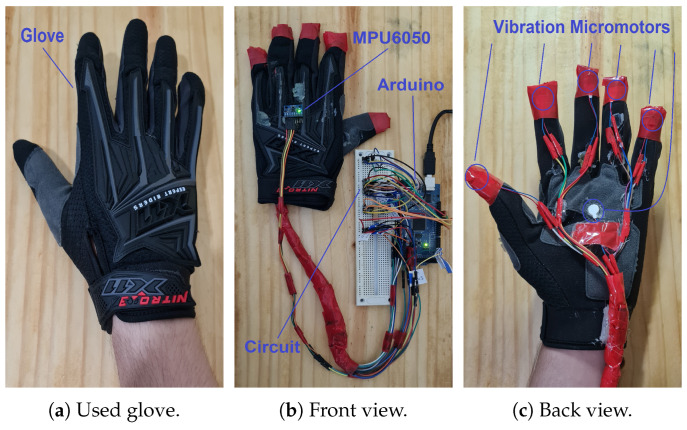
Tactile glove (*master device*).

A tactile glove shown in the Figure 2 represents the master device (MD) of the proposed platform. It has four fundamental segments, named glove (Figure 2a), IMU and microcontroller (the glove’s front view—Figure 2b), and vibration micromotors (glove’s back view—Figure 2c). The IMU is responsible for allowing teleoperation by replicating tactile glove movements on robotic manipulator. The result of the phantom omni’s movement activates micromotors in a vibration related to tactile sensation of the position that indicates object’s texture touched in a virtual way. Control of the system is conducted through an Arduino Mega 2560. Figure 2b,c shows the hardware master devices (MDH) used in this platform.

**Figure 3:**
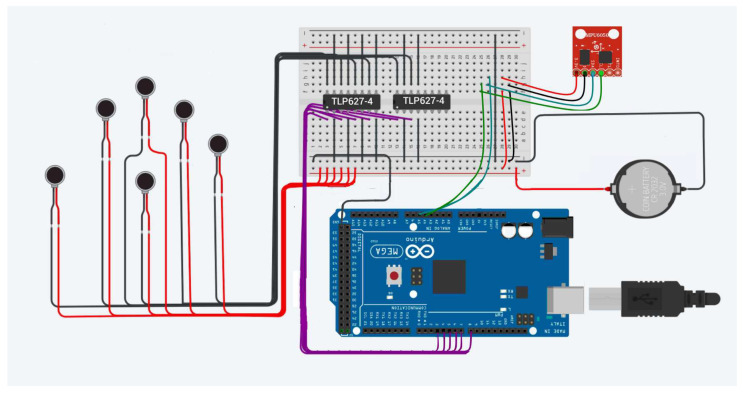
HMD Proposed Circuit.

Moreover, in Figure 3 it is possible to identify the electronic circuit which represents the master device’s hardware (MDH), which gathers the elements that execute different MD functionalities. These elements are: vibration micromotors (ROB-08449), optocouplers (TLP627-4), Arduino Mega 2560 board, MPU6050 and battery. This circuit has ATmega2560 microcontroller as its main component, since it is used to control and process the tactile glove input and output data, both in receiving signals to generate tactile sensations and sending orientation data for SD control.

The MD’s processing unit enables the correct behavior of the tactile platform. This means that the data flow through it achieves the purpose of performing remote manipulation. The microcontroller of the MDH allows the phantom omni to move replicating the tactile glove movements, and objects touched for it are felt by the operator, due to micromotors action, which are activated by the PWM signals generated in this processing unit.

As shown in Figure 3, the board controller has two component cores with different purposes. The first one, on the left in the figure, are the six ROB-08449 vibration micromotors which receive vibratory feedback representing tactile sensations. The second one, on the right, is the MPU6050 type IMU which reads the orientation of the tactile glove.

### 3.3. Communication Channel

The channel is a software entity whose purpose is to simulate a low latency connection in this platform. It is conducted by introducing a delay to data sending. The developed channel entity assigns to data exchange a fixed latency of 5ms. This value has chosen based on [6,7,8,9]. In this project, the data are Euler angles (roll, pitch, yaw), originated from MDH with destination to simulation environment.

The return data, that is to say, the tactile sensation identifier, also passes through the channel-assigned delay and it is sent from the simulation environment towards MDH. The bidirectional data exchange between MDH entity and simulation environment has the channel as a link between the two distinct platform cores, one of them referring to MD and the other to SD. The latency of the channel is equal to 10ms, considering the complete platform operation cycle.

### 3.4. Simulation Environment

The simulation environment contains SDH entity as an element for preparing data from MDH to be sent to SD, in form of joint angles ($\theta_{1}$, $\theta_{2}$, $\theta_{3}$), and it also has purpose of forwarding tactile feedback identifier, or even the absence of it, to MDH after checking SD position. SDH output data, in SD direction, seeks to move phantom omni exploring the environment tactile information under analysis. SDH data input, coming from SD, receives robotic manipulator Cartesian position information.

After inserting delay with channel entity, orientation data (roll, pitch, yaw) are received by SDH through an element dedicated to reception and initial information treatment to be used by SD control block, the SDH. Euler angles are converted for a component that returns Cartesian position (*x*, *y*, *z*) to be transformed into joint angles ($\theta_{1}$, $\theta_{2}$, $\theta_{3}$) by inverse kinematics block. SDH has inputs and outputs towards MDH, through the channel, and towards SD. The data being exchanged are, respectively, Euler angles, tactile sensation identifier, Cartesian position, and joint angles.

In this article, the SDH is configured as a model developed on Matlab-Simulink platform with components connected as can be seen in Figure 1. The blocks responsible for data arrival in SD are data reception, orientation conversion in position and inverse kinematics. Simultaneously with SD movement inside the environment, data are sent to MDH for tactile sensations simulation in MD over the elements tactile feedback identification and data sending.

**Figure 4:**
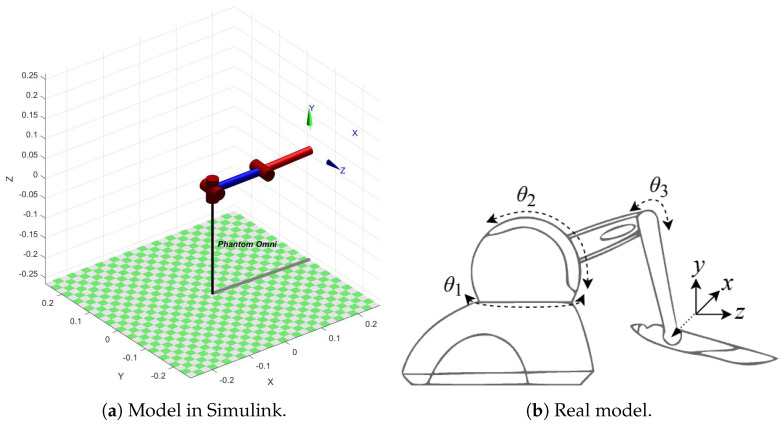
Tactile platform slave device.

The slave device proposed in this project is configured as a phantom omni robotic manipulator present in Matlab-Simulink platform through Peter Corke robotics toolbox. In Figure 4a,b it is possible to identify, respectively, the model in Simulink and the real model of the element controlled by the proposed real-time tactile platform. The phantom omni robot used has as main characteristics three degrees of freedom, 3-DoF, distributed in three rotational joints, in charge of interconnecting its links.

### 3.5. Tactile Sensations

As stated before, the vibratory feedback is responsible for generating the tactile sensations. A tactile sensation (*s*) can be expressed as a function g(·) of the variables duty cycle (*d*) and frequency (*f*) in the following way:

(1) s=g(f,d).

PWM signal frequency has the role of changing the vibration type between two possibilities: constant or with noticeable switching. These two vibration type allow to perceive the change between on and off actuators states. The constant vibrations refer to high frequencies. In this case, the micromotor vibrates at intensity imposed by apparent tension ($v_{apar}$) which is constant. Increasing in constant vibration intensity is linked to the increase of *d* and consequently of $v_{apar}$.

The $v_{apar}$ is configured as a voltage value perceived by electronic components, which is not necessarily equal to the reference voltage ($v_{ref}$), as an effect of PWM signals application. When signal frequency is high enough, voltage at vibrating micromotors terminal becomes a value between 0v and $v_{ref}$, that is, $v_{apar}$. The *d* is responsible for controlling whether components will receive influence of more or less voltage within the amplitude range for $v_{apar}$. The $v_{apar}$ calculation can be expressed as:

(2) $$v_{apar}=v_{ref}\times d.$$

Low frequencies generate vibrations with noticeable switching. This operating mode results in vibrations similar to alternating knocks. Switching constancy can be changed with a frequency fine decrease. In this case, the micromotor vibrates at intensity imposed by $v_{ref}$ at one moment and then at zero volts, that is, the alternation between on and off states becomes noticeable.

First choice to form the pair of variables referring to function g(·) should be the value of *f*, because it indicates vibration type and then *d*, since it indicates chosen vibration intensity (constant or noticeable switching). The high pulse time ($t_{high}$) serves as a manipulator element for *d*, given that its variation, conserving period (*t*), directly influences *d* percentage.

Vibration types serve as the basis for tactile sensations development. Constant vibrations simulate surfaces without irregularities, constant pulsation indicates smooth textures. Adjustments made through *d* manipulation generate different sensations with this characteristic. Vibrations with noticeable switching simulate uneven surfaces. The thumping sensation caused by evident pulsation can be manipulated to represent elements such as roughness (coarse or fine) and dripping. The creation of different tactile sensations with this characteristic can be conducted over changes in *f*.

Constant vibrations have their intensity varied by changes in $v_{apar}$, caused by *d* changes. In vibrations with noticeable switching, *d* variation does not affect $v_{apar}$, but the vibration time remains under $v_{ref}$ influence before it becomes zero volts. The $v_{ref}$ used in platform’s ROB-08449 micromotors is 3.3v of Arduino Mega 2560 board.

The *f* value, which is the threshold between two operating modes (constant and perceptible switching), could be found experimentally, applying tests with vibration micromotors.

In experiments carried out for this paper, *f* values were assigned to a given *d* and the occurrence of a change in vibration type was verified. By using this technique with ROB-08449 micromotors of the proposed tactile platform, it was realized in a practical way, that the threshold value is 50 Hz.

The threshold main characteristics between vibration forms are *f* of 50 Hz and *t* of 20 ms. The developed experiment has used *d* of 50% during tests. The experiment started with 12.5 Hz and 80 ms of *f* and *t*, respectively. In this configuration resulting vibration type was noticeable switching. Tests continued to cut *t* value in half and doubled *f* at each step. At the end of the experiment, the relationship between spacing decrease between switches and changes made in PWM signal parameters (*f* and *t*) became evident. According to perceived results, constant vibrations are given by f>50 Hz. Vibrations with noticeable switching are generated from *f*, with the following characteristic: f<50 Hz.

When the vibration type chosen is perceptible switching, *d* excessive increase causes vibration to become constant. High *d* values make $t_{low}$ excessively short, mischaracterizing the switch. In developing tactile sensations, *d* is used to change chosen vibration mode intensity. In vibrations, which generate beats sensations, *d* changes switch intensity. However, it can also change the vibration (switched to constant) type. It was found experimentally that this change threshold is given by d=97.5%.

The developed experiment tried to observe changing *d* effects on switched vibration. These effects range from impacts on changing time between switches to making them imperceptible. In the test’s initial configuration, the switched vibration frequency that would be part of all experiment stages was chosen, being f=12.5 Hz. At first, the duty cycle value was chosen as d=50%. Following values were d=75% and d=93.75%. After the last step, changes were made to *d* value through fine tuning. Changing in vibration type (switched to constant) occurred with the following duty cycle value: d=97.5%.

**Figure 5:**
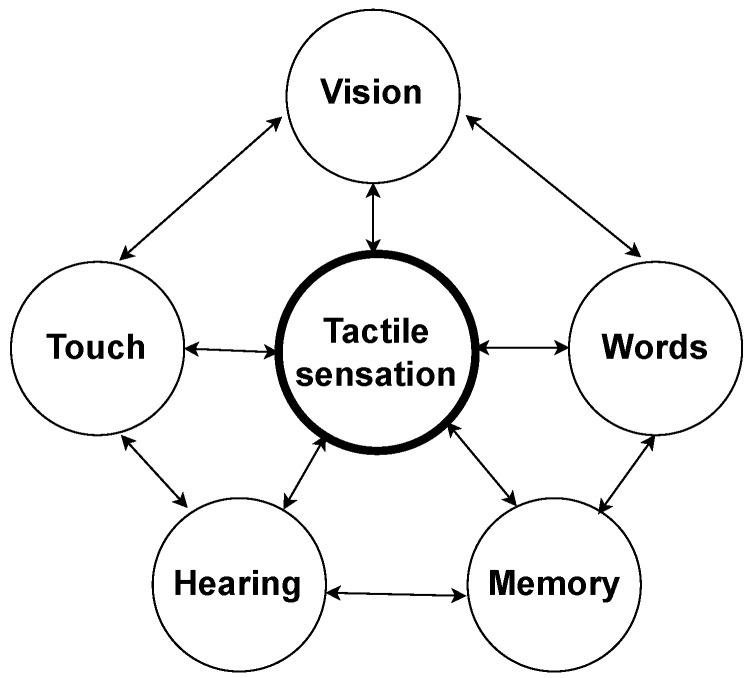
Parameters for extracting tactile sensations features.

Tactile sensations modeling in this article was similar to the methodology presented in [24]. Thus, the touch sensations understanding happened by the parameters present in Figure 5 and their characteristics were associated with vibration patterns through PWM signals manipulation.

Considering vibrations with perceptible switching, an operation mode enabled by signals with f<50 Hz. In the same *f*, switching occurrence constancy does not change, regardless of the change in other PWM signal aspects. The *d* variation influences intensity (force) in which knock sensations are perceived. Experimentally, it was identified that frequency range 11.11 Hz <f<50 Hz is responsible for generating switching, considerably close, resulting in small granules sensation. The *f* increase and decrease within aforementioned range, decreases irregularity sensation thickness and increases, respectively. In range 3.33 Hz <f<11.11 Hz beat sensations are still close and simulate thick granules. On the other hand, frequencies within range f<3.33 Hz generate considerably spaced switching. In the case of d<25% vibrations in perceptible switching mode within this range are assigned softness.

**Figure 6:**
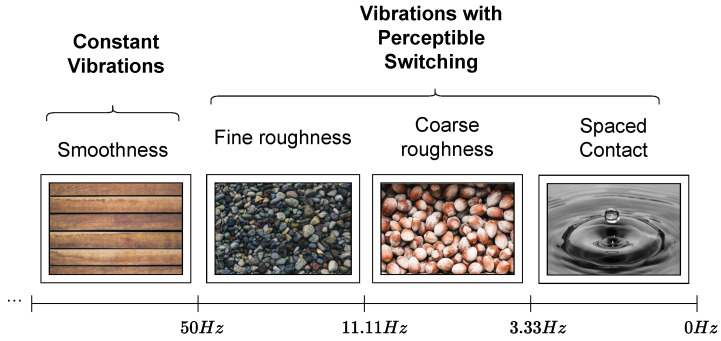
Association among tactile sensations, vibrations and frequency ranges.

Considering constant vibrations, an operation mode enabled by signals with f>50 Hz. The tactile sensations, *s*, possible to generate with this vibration characteristic depend on *d* variation, that is, any *f* within aforementioned range can be chosen. Constant vibrations represent smooth surfaces and in this case d<25% indicates smooth textures. The *d* increase is proportional to stiffness increase. Figure 6 shows a summary of possible sensations types generated through different vibrations and the respective frequency bands.

In the vibratory feedback intensity change due to *d*, it is useful to improve tactile sensations perception. When simulating surfaces with irregularities, PWM signal $t_{high}$, which is different from the change caused by *d*, is configured as relief (irregularity) width and $t_{low}$ as spacing between them. Changing granules type present on the surface can be conducted by varying *f* within range 11, 11Hz<f<50Hz to generate fine irregularities tactile sensations or in range 3.33Hz<f<11.11Hz in order to produce coarse irregularities sensations.

PWM signals with frequencies f<3.33Hz simulate spaced contact tactile sensations. In this kind of sensation, interactions with objects generate switches with occurrences, relatively distant from each other, they do not represent irregularities. This sort of signal indicates sensations similar to elements falling continuously on a contact surface.

Constant vibrations simulate smooth surfaces and in these cases *d* is responsible for varying how hard or soft they should be. The parameters for PWM signals to generate constant vibrations that simulate smooth surfaces are f>50Hz and d<25%.

Using the same range of *f* and an inverted range of *d* it is possible to produce hard surface sensations. The parameters for this sort of vibration are f>50Hz and d>25%. The stiffness and softness degree are regulated by *d*, and within the previously indicated ranges, different levels of these two tactile sensation characteristics resulting from constant vibrations are generated.

## 4. Results

**Figure 7:**
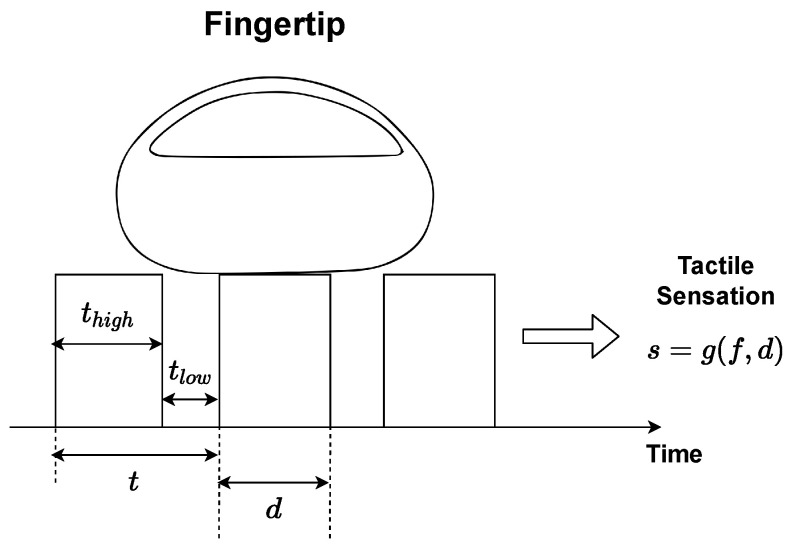
Tactile sensations of virtual textures, perceived through vibratory stimulus.

As shown in Figure 7, the PWM signal characteristics generate vibrations responsible for producing tactile sensations transmitted to tactile platform users. These tactile sensations allow associations between perceived sensations and different textures virtually touched.

The Table 1 presents PWM signal parameters that generate each of the tactile sensations modeled for this paper.

**Figure 8:**
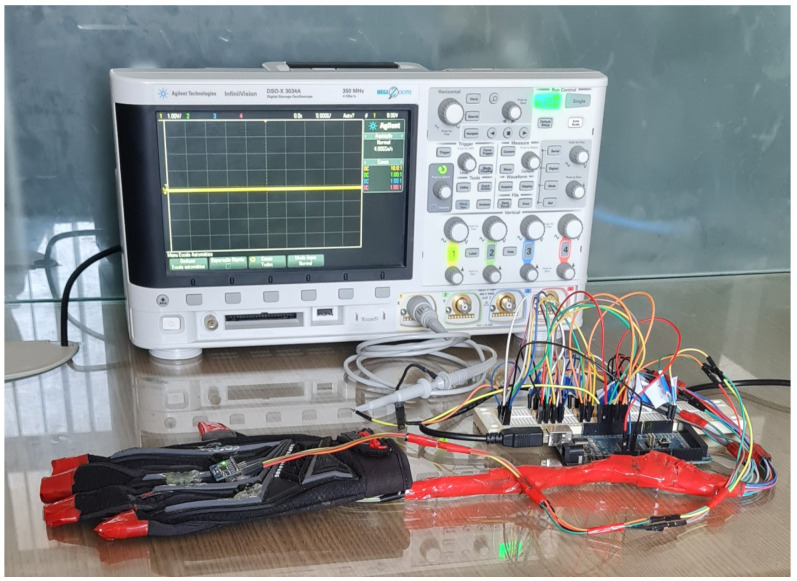
Configuration for viewing PWM signals related to tactile sensations.

The PWM signals associated with modeled tactile sensations, *s*, were analyzed using an oscilloscope as shown in Figure 8. PWM signals visualization helps to understand the relationship between their characteristics and the textures details they represent in form of tactile sensation that must be transmitted to platform operator’s glove.

**Figure 9:**
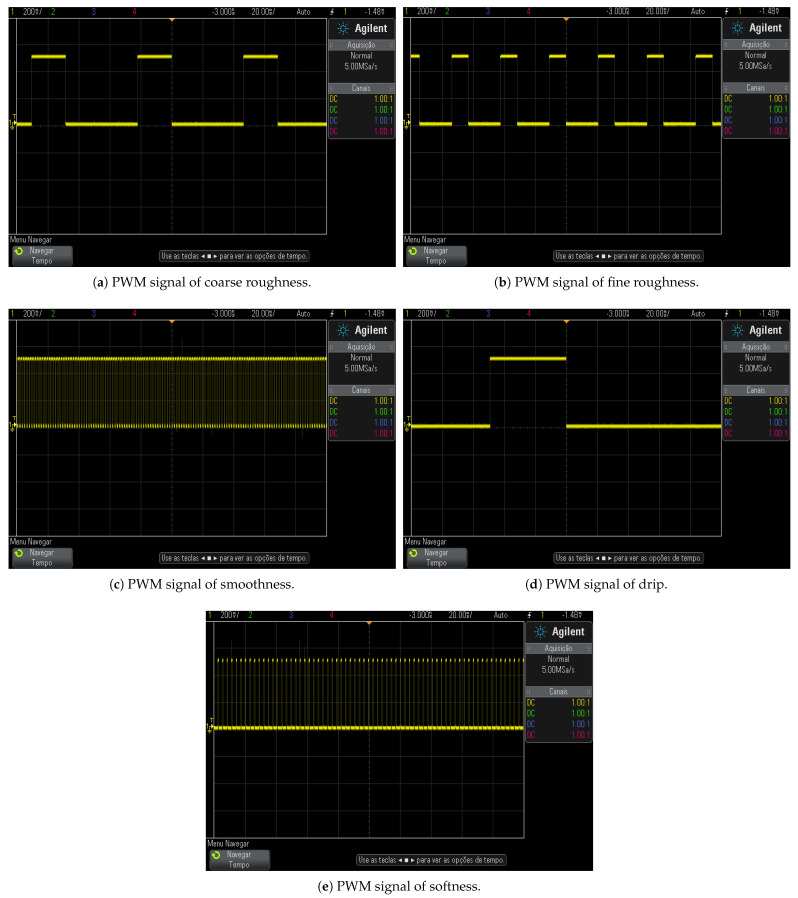
PWM signals of tactile sensations.

Coarse and fine roughness sensations as present in Figure 9a,b, respectively, have similar frequencies PWM signals, however different enough to make a pulse rate perceptible difference. In coarse roughness the vibration simulates spaced beats sensations with the following characteristics: 3.33Hz<f<11.11Hz and s=g(7.2Hz;32%), in such a way that actuator high pulse time, $t_{high}$, indicates the granules (irregularity) size present on virtual surface, being greater than the fine roughness, whose characteristics are described by 11.11Hz<f<50Hz and s=g(15.6Hz;34%). The coarse and fine roughness tactile sensations are similar to contact with different thicknesses boulders.

Smooth textures are simulated by constant vibrations. The characteristics of which are f>50Hz, d>25% and s=g(333Hz,67%). This sort of signal does not represent bumps caused by noticeable switching, since the vibration is uniform. According to Figure 9c, smoothness tactile sensation PWM signal has a value of *f* and *d* high enough to generate a constant vibration that results in the simulation of a soft and rigid surface. The modeled smooth sensation indicates contact with materials similar to polished wood.

Dripping sensation has a low frequency, as described by the characteristics f<3.33 Hz and s=g(1.7Hz;17%), which introduce switching effect on vibration. This lack of uniformity produces spaced contact sensation in order to simulate continuous interaction with droplets. This simulated characteristic can be perceived by the PWM signal, responsible for generating sensation of dripping, since the pulses are sufficiently spaced for this as presented in Figure 9d.

The softness feeling is intended to simulate textures, in which the contact with virtual surfaces is light, through weak pulsations that have short $t_{high}$ and high *f* according to f>50Hz, d<25% and s=g(166.7Hz;16.7%). In order to generate this kind of surface, the vibration occurs by a PWM signal, present in Figure 9e, with a small value of *t*, mostly in $t_{low}$. These characteristics generate a sensation of constant vibration with low intensity, that is, a low value of *d*. The softness tactile sensation, simulated in this article, is similar to the experience of running fingers over cotton.

**Figure 10:**
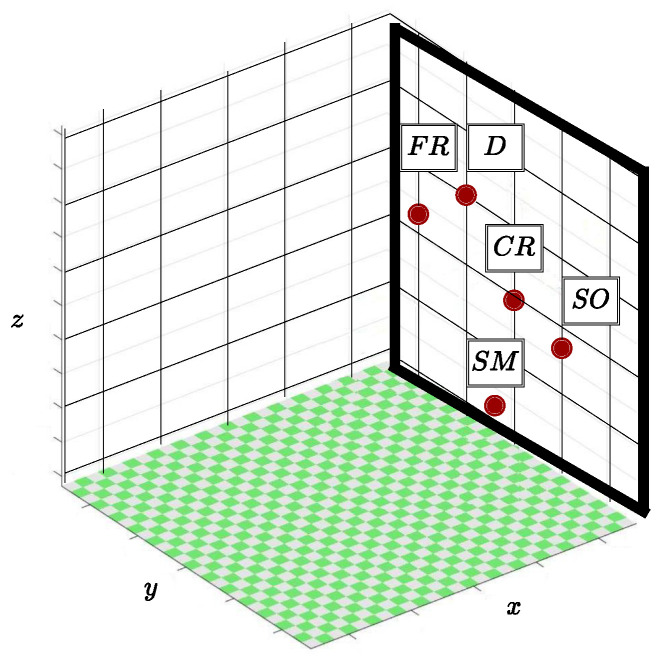
Plane with the referring regions to the textures.

In Figure 10 it is possible to identify the plane of the environment in which SD is inserted, formed by axes *z* and *y* that works as a generating tactile feedback wall. That is, when phantom omni’s tool touches specific points on this wall, sensations related to different textures are simulated. As shown in Figure 10, there are five regions related to each of the tactile sensations developed, coarse roughness (CR), fine roughness (FR), smoothness (SM), dripping (D), and softness (SO). Contact with these positions is enough to stimulate activation of the actuators present in MD, responsible for simulating interaction with different textures. External movements in regions referring to tactile returns result in deactivation of the vibration micromotors and thus represents absence of interaction with texture regions.

**Figure 11:**
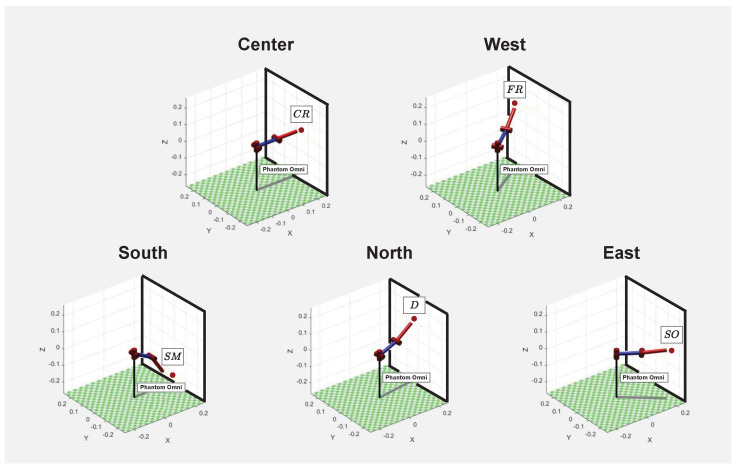
SD contact with texture regions.

Figure 11 shows the directions in which SD tool points in order to reach the regions related to different textures. Each of these points present in Figure 10, as associated by Table 2, belong to one of the directions, that is, if the phantom omni touches the plane centered position under analysis, the feedback simulated will be coarse roughness. Movements in the west direction make it possible to reach the region referring to fine roughness texture. By taking SD tool towards south direction of the *z* and *y* plane, in the explored environment, it becomes possible to return smooth tactile sensation. Lifting the robotic manipulator until the highest plane point, the north direction is reached, which contains texture related to dripping sensation. Finally, in the east direction, the region referring to the softness sensation is present. Some videos showing how the platform works in real-time can be found at https://drive.google.com/drive/folders/19SY-YG28X7QefgerhnByHA8I60ZiBIoA (accessed on 25 November 2022).

## 5. Conclusions

This article presented a real-time test platform development for tactile internet applications. The platform allows tactile interaction between a human operator, using a tactile glove, with different textures objects, through a phantom omni-type manipulator robot, simulated on the Matlab-Simulink platform.

The results show that the platform allows simulating several sensations types, enabling the test of various algorithms and techniques associated with tactile internet. The test platform proposed here can significantly contribute to the development of new strategies used to minimize problems associated with tactile internet.

In a future work, modifications will be made in elements such as: master device’s hardware, simulation environment and communication channel. Inserting a MPU9050 and exchanging Arduino Mega 2560 board for Arduino Lilypad 328, that is specific to wearable applications, certainly will improve these results. The introduction of a real phantom omni will bring the possibility of physical interaction with objects, during tactile exploration. Moreover, a modification in the channel simulation introducing connection variable latency would increase the realism of the tests carried out by the platform.

## Figures and Tables

**Table 1:** Modeled tactile sensations.

Tactile Sensations	$t_{\mathrm{high}}$ (ms)	$t_{\mathrm{low}}$ (ms)	*d* (%)	*f* (Hz)
Coarse roughness	45	94	32	7.2
Fine roughness	22	42	34	15.6
Smoothness	2	1	67	333
Drip	100	500	17	1.7
Softness	1	5	16.7	166.7

**Table 2:** Textures’ location and identifier.

Direction	Texture	*x*	*y*	*z*	Id
Center	Coarse roughness	0.1	0.1	2.09	1
West	Fine roughness	−0.6	0.3	0.79	2
South	Smoothness	0.5	−0.85	1.46	3
North	Drip	0.34	0.62	2.09	4
East	Softness	2.07	0.27	2.29	5

## Data Availability

Not applicable.
